# Correlation of balance posturographic parameters during quiet standing with the berg balance scale in patients with parkinson’s disease

**DOI:** 10.1186/s12883-023-03386-1

**Published:** 2023-10-06

**Authors:** Wei Bao, Yuyan Tan, Ying Yang, Kai Chen, Jun Liu

**Affiliations:** 1 School of Mechanical Engineering, Hangzhou Dianzi University, Hangzhou, 310018 Zhejiang China; 2 Department of Neurology, Institute of Neurology, Ruijin Hospital, Shanghai Jiao Tong University School of Medicine, Shanghai, 200025 China

**Keywords:** Parkinson’s disease (PD), Balance control, Center of pressure (COP), Posturography, Berg Balance Scale

## Abstract

**Background:**

Parkinson’s disease (PD) is often clinically associated with posture instability and more easily falling. The Berg balance scale is a clinical indicator commonly used to subjectively evaluate a patient’s balance ability. Meanwhile, computerized force platforms have been used in research on postural control. The various parameters obtained from posturography are interpreted to assess balance ability. The present study aims to explore the correlations between posturographic variables and the BBS, and furthermore to efficiently evaluate postural instability and fall risk of early and moderate PD patients.

**Methods:**

A total of 46 PD patients were involved in the experiment. Patients were asked to perform BBS tests and force platform tests under eye open (EO) and eye closed (EC) conditions. The recorded COP signal was analyzed with the time domain statistical method, the frequency domain method of Power Spectral Density (PSD), and structural methods of Stabilogram Diffusion Analysis (SDA), Sway Density Plot (SDP) to retrieve different posturographic variables. The correlation between posturographic variables under EO and EC conditions with BBS was compared statistically. The significantly correlated posturographic parameters were then applied to analyze posturographic differences between different groups: faller vs. non-faller (patients with/without a history of falls in the past 12 months).

**Results:**

Among the different posturographic parameters, the prediction ellipse area, the slope of the regression line at a high-frequency band of PSD in the medial-lateral (ML) direction, the crossover point of the regression lines of SDA in the anterior-posterior (AP) direction, and the distance between successive peaks of SDP had significant correlations with BBS. These selected BBS-related parameters also showed significant differences between faller and non-faller. The selected posturographic parameters can be used as effective indicators to evaluate the balance ability of Parkinson’s disease patients.

**Supplementary Information:**

The online version contains supplementary material available at 10.1186/s12883-023-03386-1.

## Introduction

Parkinson’s disease (PD), is a neurodegenerative disease clinically characterized by symptoms such as static tremoring, mobility delays, reduced voluntary movement, posture and balance disorders, and freeze of gait [1]. Posture and balance disorders can predispose patients to fall due to postural instability [2]. The postural instability in people with PD is attributed to impairments in many aspects of balance control including: rigidity affecting biomechanics, bradykinesia of postural responses, anticipatory postural adjustments, impaired kinesthesia for sensory integration, bradykinetic gait with freezing, and less automaticity of gait and balance [3, 4]. It is thus very important to evaluate the balance ability of PD patients accurately when standing [5].

At present, clinical rating scales, such as Berg Balance Scale (BBS) are commonly adopted as clinical methods to evaluate patients’ balance ability [6, 7]. BBS is originally designed to assess the risk of falling in elderly patient. It is divided into 14 items, which mainly evaluates the human body’s standing balance ability. The full score of the BBS is 56, and a higher score indicates a better human balance control. Evaluation of BBS provided an important basis for follow-up evaluation of patient’s treatment status. BBS has been reported to provide a reliable and valid assessment of patients with PD disease, and can be used to assess the clinical conditions for appropriate treatments [8–10]. BBS is reported to be able to differentiate between people with Parkinson’s disease with or without a history of falling in a moderate effect, and be able to predict the fear of falling in people with Parkinson’s disease during the drug off-phase [11]. However, the observation scaling methods are often carried out after notable symptoms. Several studies highlighted abnormal postural sway in the early stage of PD before the onset of clinical symptoms [12].

Postural instability can be quantitatively assessed under open and closed eyes conditions using force platforms, which provide a more objective and sensitive measurement than clinical scales, despite the different intra-subject variability of the computed posturographic parameters [13–15]. The center of pressure (COP) data can be further processed to achieve posturographic parameters. The characterization of posturographic parameters can be categorized into two types: global parameters, which are mainly used to evaluate the overall size of postural sway, and structural parameters, which decompose the postural sway into elements and identify the relationship between each element. Global parameters are mainly retrieved by calculating the time domain of the COP trajectory. Power spectral density (PSD) analysis is applied to analyze different frequency bands and some typical frequency points in the spectral of COP signal [16]. It helps to find sensitivities of complex attributes associated with physiological systems and leads to detect and discriminate postural control system impairments [17]. With regard to structural parameters, stabilogram diffusion analysis (SDA) is a commonly applied method [18–20]. SDA describes the signal as persistent when there is a positive correlation with time and anti-persistent when there is a negative correlation with time. A sway density plot (SDP) has also been proposed as an effective method to correlate with the anticipatory active torque by calculating the peak and valley sizes in the SDP [21, 22].

Time domain parameters have been used to evaluate postural stability in PD patients in many studies. The change in body sway can be described by many variables, such as area, velocity, path, root mean square and standard deviation of COP [23]. PD patients, evaluated in upright standing under open and closed eyes conditions, show greater spontaneous sway than elderly people without neurodegenerative disorders [24]. It is also found that there was a significant difference in the average speed of COP signals between PD patients and age-matched controls [25]. The average values of sway range both in the AP and ML directions measured in the PD group during quiet standing (QS) are significantly higher than those in the control group [26]. Jazaeri et al., reported greater path length in PD patients than healthy subjects [27]. Johnson et al. studied the relationship between posturographic variables and BBS and found that the sway area was significantly and positively correlated with BBS [28]. Ferrazzoli et al. also showed that the BBS score was significantly negatively correlated with COP standard deviation (SD) in the ML direction, as well as sway area [29]. In the study of Barbosa et al., there is only a weak correlation between the SD of the COP and BBS in the open-eye state [25]. However, these time domain parameters of the COP trajectory reveal few insights into postural control mechanisms.

Comparatively, frequency domain global parameters and structural parameters are hypothesized to reveal the control mechanism of human body balance [22, 30, 31]. Slopes of the low and high frequency bands region of Power spectral density (PSD) analysis are used to discriminate between PD and healthy individuals. By using the PSD method, groups of PD patients with respect to healthy individuals can be identified in Yamamoto et al.’s report [32]. Mitchell et al. applied stabilogram diffusion analysis (SDA) to evaluate posturographic parameters between PD patients and healthy elderly controls and found that PD patients were characterized by an increase in postural sway in the ML direction [33]. A sway density plot (SDP) is also effective in distinguishing between healthy and PD patients [34]. However, there have been few studies on balance grading among PD patients using these methods.

Since the characteristic variables obtained from COP data reveal the postural control ability, we hypothesize that these variables may correlate with BBS in PD. The current study aims to characterize the PD patients’ posturography in quiet standing through a comprehensive analysis of COP data with global and structural variables, and to correlate these variables with the BBS total score. By correlation, optimal posturographic variables can be retrieved to evaluate the balance ability of PD patients, and the underlying balance control mechanisms in PD patients can be explored.

## Materials and methods

### Participants and berg balance scale tests

A total of 46 patients, including 24 males and 22 females, with an average age of 68.2 ± 7.1 years, participated in the experiment. The PD patients had baseline characteristics with a disease duration of 3.96 ± 1.84 (Table 1). The mean Hoehn-Yahr (H&Y) scale stage was 2.17 ± 0.64, indicating mild-to-moderate bilateral disease with some postural instability, but still physically independent. The mean UPDRS-III sub-score was 21.9 ± 8.8. Other information such as age, gender, height, weight, BMI and MMSE were listed in Table 1. All participants were first confirmed to have PD by physical examination, and patients who were unconscious or had a history of surgery were excluded from the study. PD was diagnosed by senior movement disorder specialists based on Movement Disorder Society (MDS) diagnostic criteria [35]. During the diagnosis, BBS [6], Hoehn and Yahr (H&Y) scale [36], and MDS-Unified Parkinson Disease Rating Scale (MDS-UPDRS) assessments were also performed [37]. Other examinations such as Minimum Mental State Examination (MMSE) [38], Hamilton Depression Scale (HAMD-17) [39], Gait and Falls Questionnaire (GFQ) [40], Freezing of Gait Questionnaire (FOG) [41], 39 Questionnaires on quality of life in Parkinson’s disease (PDQ-39) [42], Hamilton Anxiety Scale (HAMA) [43], Montreal Cognitive Assessment Beijing version (MOCA) [44] were also recorded [44]. The inclusion criteria was in accordance with the MDS’s revised clinical diagnostic for Parkinson’s disease (2015 edition) and was as follows: The PD patients were generally in good physical condition; age less than 85 years, more than 40 years old, Class1 ≤ H&Y grading ≤ 3; Medication regimen was kept unchanged during the study. Patients with a history of falls in the past 12 months were also included. A faller was defined as an event which results in a person coming to rest inadvertently on the ground, floor, or other lower level [45]. The exclusion criteria was as follows: a history of severe neurological and psychiatric disorders, patients with significant cognitive impairment (MMSE < 24) or inability to complete the questionnaire independently, severe medical conditions preventing the patient from completing the experiment, existing implantable materials for human implants such as intracranial stents, pacemakers, coronary stents, and cochlear implants; pregnant or lactating women; secondary causes (inflammatory, drug-induced, vascular and toxin-induced parkinsonism, etc.), parkinsonism with other neurodegenerative diseases (progressive supranuclear palsy, multiple system atrophy, cortical basal ganglia degeneration, Wilson’s disease, etc.), other neurological diseases such as stroke, anxiety. All subjects provided informed written consent prior to participation in the study. This study was conducted in accordance with the guidelines of the World Medical Association Declaration of Helsinki (2000) and was approved and supervised by the Ethics Committee of Shanghai Ruijin Hospital (Approval Number: LWEC2019017). After a detailed description of the experiment, all participants signed informed consent forms. The clinical status of the patients and their measured BBS scores, H&Y scales and MDS-UPDRS are shown in Table 1. All participants had independent experiment numbers, which were used to identify the data collection during and after the experiment. The experimental data of patients were collected from December 2019 to November 2020 within the permitted time.

**Table 1:** Demographic data and Scales for PD group (N = 46)

**No.**	**Age/g**	**D(Y)**	**H&Y**		**M-UP**	**BBS**	**No.**	**Age/g**		**D(Y)**	**H&Y**	**MDS**	**BBS**
1	60/F	9	2.5		60	56	24	57/F		5	2.5	93	43
2	64/F	3	1		22	56	25	64/M		3	2.5	64	45
3	65/M	3	2.5		40	56	26 ^Fa^	65/M		6	2.5	98	51
4	60/F	8	1		37	56	27	63/F		9	2	72	49
5	64/M	2.5	2		34	56	28	69/F		3	3	68	29
6	65/M	7	1		30	56	29	65/M		4	2	75	49
7	70/M	3	2		30	56	30	60/M		2	2	71	48
8	64/M	5	1		24	56	31	64/F		3.5	3	60	37
9	55/F	5	1		11	56	32 ^Fa^	65/M		5	3	123	37
10	50/F	3	2		53	55	33	52/M		5	2	46	47
11	68/M	6	1.5		22	55	34	65/M		3	2	43	47
12	49/F	3	2.5		66	49	35	65/M		3	2.5	61	47
13	70/M	4	2.5		46	49	36	64/M		2	2	70	45
14	65/M	5	2.5		76	55	37^Fa^	65/F		2	2	86	40
15	60/M	2	1.5		49	55	38 ^Fa^	68/M		5	3	83	39
16	61/F	3	2		54	55	39	56/F		2	3	72	38
17	65/M	2	1.5		32	54	40 ^Fa^	75/F		3	3	90	35
18	60/M	3	2		64	54	41	69/F		3	1.5	48	35
19	64/F	5	2		62	54	42 ^Fa^	66/M		3	3	94	33
20	64/F	8	2		44	54	43 ^Fa^	70/M		4	3	100	33
21	56/F	3	1.5		46	53	44 ^Fa^	60/Fe		4	3	73	29
22	64/F	3	2.5		58	45	45 ^Fa^	64/Fe		5	3	74	25
23	65/M	3	1.5		74	51	46 ^Fa^	65/Fe		5	3	108	22
				**(mean ± SD)**					**(Min,Max)**				
Age(Y)				68.2 ± 7.1					(49,75)				
Disease duration (Y)				3.96 ± 1.84					(2,9)				
Sex (% Female)				22(47.8%)					/				
Body weight (kg)				64.1 ± 11.74					(45,88)				
Height (cm)				165.6 ± 6.25					(152,177)				
Body mass index				23.5 ± 4.2					(16,30)				
H&Y score				2.17 ± 0.64					(1,3)				
MDS-UPDRS total				61 ± 24.75					(11,123)				
MDS-UPDRS score- III				21.9 ± 8.8					(4,44)				
Berg Balance Scale				48.2 ± 9.1					(22,56)				
HAMD-17				13.21 ± 7.69					(3,31)				
FOG				11.97 ± 4					(3,19)				
PQD-39				37.47 ± 25.4					(7,80)				
HAMA				16.86 ± 7.49					(4,29)				
MOCA				21.4 ± 4.14					(14,28)				
MMSE				25.93 ± 1.86					(24,29)				
GFQ				19.04 ± 9.42					(3,48)				
phenotype (tre/PIGD)				20/26					/				
Faller/non-Faller				10/36					/				

PD: Parkinson disease dataset; BBS: Berg balance scale; * indicates patients treated with levodopa; H&Y: Hoehn-Yahr Scale; M-UP: MDS-Unified Parkinson’s Disease Rating Scale; HAMD-17: Hamilton Depression Scale-17; FOG: Freezing of Gait Questionnaire; PQD-39: Quality of life questionnaire for Parkinson’s patients; HAMA: Hamilton Anxiety Scale; MOCA: Montreal Cognitive Assessment; MMSE: Mini-Mental State Examination; GFQ: Freeze gait questionnaire; tre: tremor-dominant; PIGD: Postural instability and gait disorder; SD: standard deviations; D (Y):Disease Duration (year); M:male; F:female; upper corner marked Fa: patient with a history of falls within 12 month; g: gender

### Measurements of the center of pressure

**Fig. 1:**
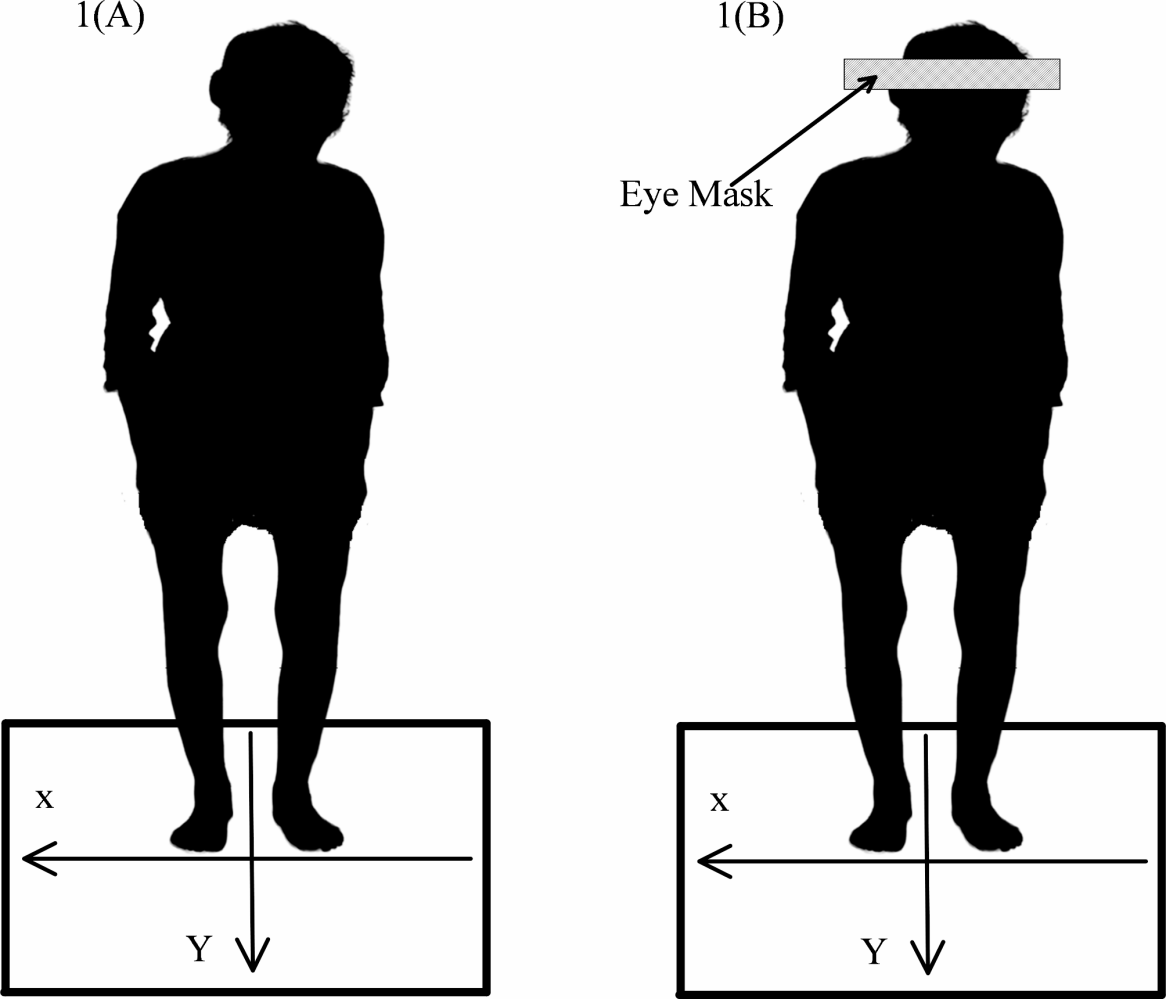
A barefoot patient standing still on a force platform, (**A**) EO state, (**B**) EC state. EO = Eye Open, EC = Eye Close, X = ML direction, Y = AP direction

In this study, the force platform (Model BP400600, AMTI, USA) was used to retrieve patients’ COP time series. The sampling frequency of the force measuring system was 500 Hz. According to the position of patients standing, anterior-posterior (AP) was noted as the *y* direction, medial-lateral (ML) was noted as the *x* direction, and the coordinate origin was the central point of the platform. Before the experiment, participants were informed of the test procedure. As shown in Fig. 1, participants were required to stand still on the force platform with bare feet, and shoulder width apart and hands hanging naturally on each side of the body.

Before each measurement, the range of the distance between heels was reconfirmed within 20 ± 3 cm, and the range of angles of the foot with respect to AP axis was also reconfirmed within 20 ± 2 degrees. Their eyes were focused on a black dot on the wall 3 m in front. The participants were tested under two states: open-eye state (EO), as shown in Fig. 1A, and closed-eye state (EC), as shown in Fig. 1B. In the EC state, the patient was required to wear an eye mask. Patients were tested for 60 s each time, and the same process was repeated three times with a 5-minute rest between each time. During the 5-minute rest, the subjects were required to sit on a comfortable sofa to relax and recover their physical strength.

### Analysis of the COP parameters

The time series COP data were collected at 500 Hz sampling rate and were processed offline using the Matlab® software with a 10 Hz, fourth-order, low-pass Butterworth filter. After that, the posturographic parameters (refer to Table 2) were calculated through customed MATLAB® algorithms. As the starting point of each measurement was different, the average value of COP displacements was taken as the offset value. The actual offset of COP positions in the *x* and *y* directions were removed before calculation. The difference in the average value of the COP displacements between patients was in the range of 2 to 40 mm. In this study, global and structural parameters were calculated as shown in Table 2. The time domain global variables are commonly applied to quantify the stabilogram during quiet standing, and their definitions were explained in various literature. The definitions of the frequency domain variables and the structural variables are illustrated in the following sections. The calculation methods of the listed parameters are provided in the supplemental material of the current paper.

**Table 2:** Global and structural posturographic parameters

Category	Parameters	Description
	*COP* *_* *v*	Average value of COP velocity in ML or AP direction
Global (Time domain)	*COP_SD*	Standard deviation of COP position in ML or AP direction
	*COP_PEA*	Prediction ellipse area that encompasses 95% of COP points
	*COP_Rg*	Range of COP position in ML or AP direction
	*COP_SP*	Sway Path of COP position in ML or AP direction
Global (Frequency domain)	*PSD_FB80*	Integrated area of PSD at the frequency band that contains up to 80% of PSD in ML or AP direction
	*PSD_LPeak*	Peak-value of PSD at a low-frequency band (0.01–0.5 Hz) in ML or AP direction
	*PSD_HPeak*	Peak-value of PSD at a high-frequency band (0.5-1.2 Hz) in ML or AP direction
	*PSD_LSlope*	Slope of the PSD regional regression line at a low-frequency band (0.01–0.5 Hz) in ML or AP direction
	*PSD_HSlope*	Slope of the PSD regional regression line at a high-frequency band (0.5-1.2 Hz) in ML or AP direction
	*PSD_CP*	Crossover point of the two PSD regional regression lines
Structural	*SDA_SSlope* *SDA_LSlope*	Slope of stabilogram at short-term region on log-scale SDA
		Slope of stabilogram at long-term region on log-scale SDA
	*SDA* _ *CP*	Crossover point of the short-term and long-term lines regional regression lines
	*SDP* _ *MT*	Population mean value of the time interval between successive peaks (MT) in sway density plot
	*SDP* *_* *MP*	Population mean value of the peaks (MP) in the sway density plot
	*SDP* *_* *MD*	Population mean value of the distance between successive peaks (MD) in the sway density plot

### Analysis with power spectral density

**Fig. 2:**
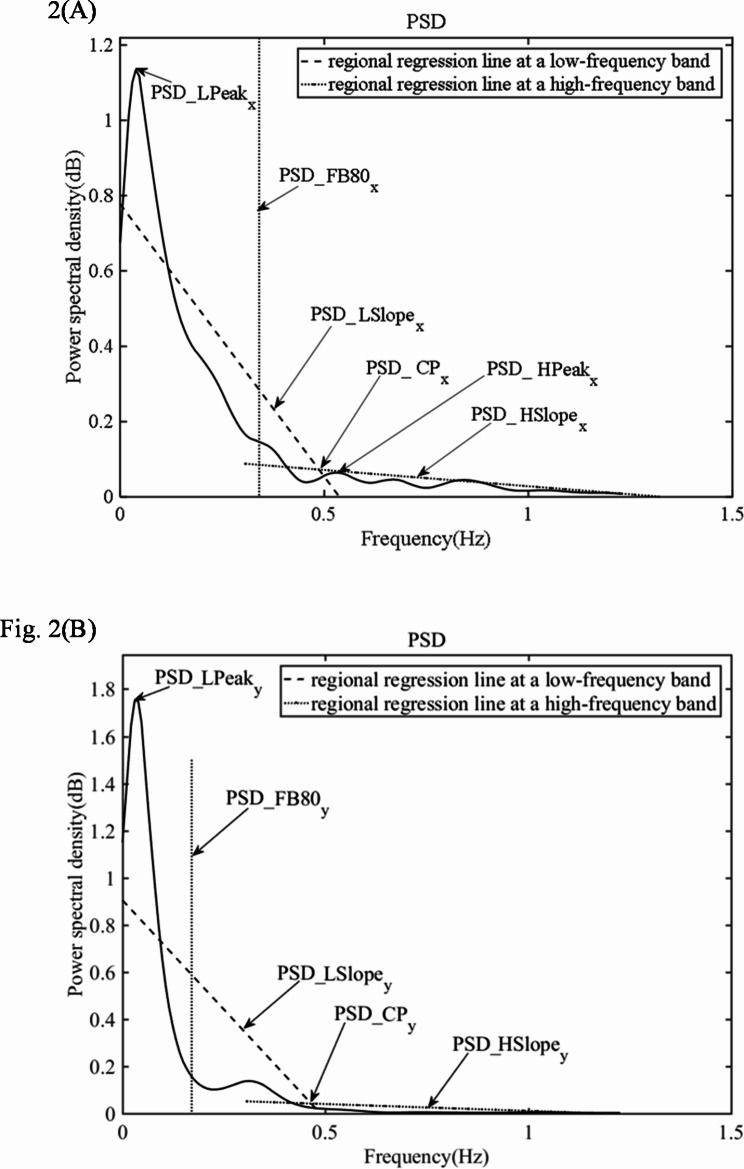
PSD analysis diagram of a sample PD patient’s COP signal at the EO state: (**A**) in the ML direction; (**B**) in the AP direction. PSD = Power Spectral Density, ML = medial-lateral, AP = anterior-posterior, x = ML, y = AP.

Power spectral density (PSD) represents the strength variation of the signal as a function of frequency. The PSD was calculated by the fast Fourier transform (FFT) method [16]. The PSD values can be categorized into a low frequency band of 0.01–0.5 Hz and a high frequency band of 0.5–1.2 Hz [16]. The peak value of PSD and frequency band width of COP signals that contained classical values of 80% of PSD were calculated. Slopes of the regions under the two different frequency bands and their crossover points were calculated. Figure 2 shows PSD changes with the frequency of COP position in a sample PD patient in the ML and AP directions at the EO state.

### Stabilogram diffusion analysis

**Fig. 3:**
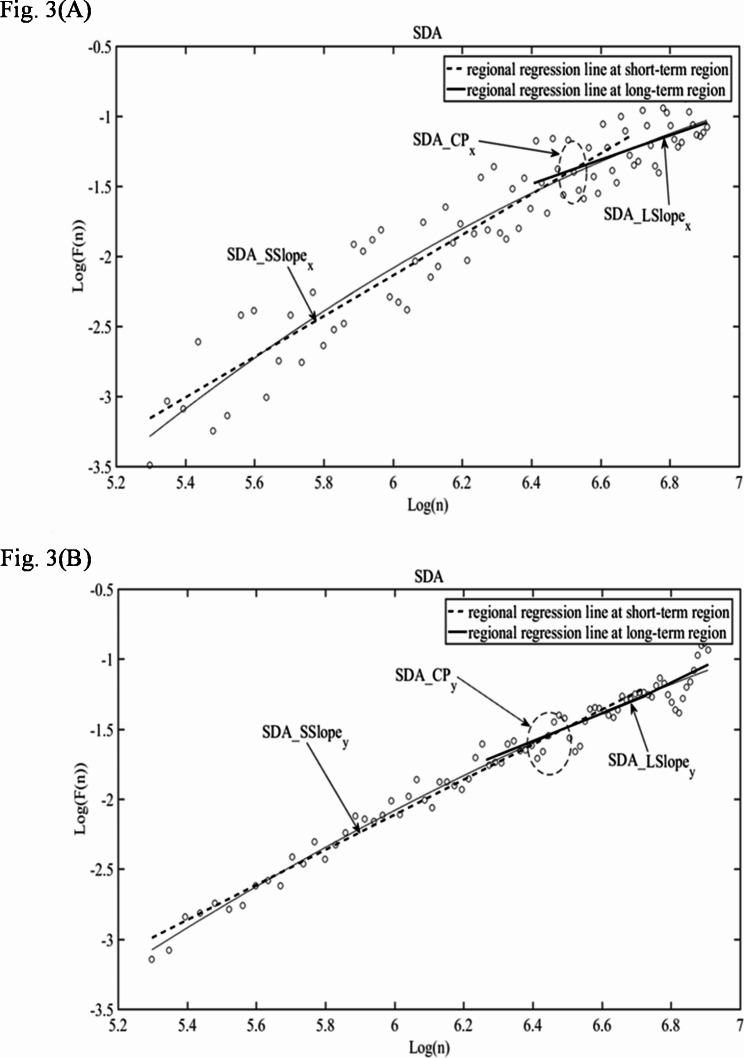
Logarithmic diffusion diagrams generated by SDA analysis of a PD patient’s COP signal at the EO: (**A**) in the ML direction; (**B**) in the AP direction. SDA = Stabilogram Diffusion Analysis, ML = medial-lateral, AP = anterior-posterior, x = ML, y = AP.

The stabilogram diffusion analysis (SDA) method is a quantitative statistical measure of the apparent random variations in COP trajectories. The COP velocity signal is decomposed into different time scale parts [30]. One part is a short-term region, and another part is a longer-term region. The short-term region indicates persistence, while the long-term region represents anti-persistence. The transition point from persistence to anti-persistence is called the crossover point. The values of the short-term scale index, long-term scale index, and crossover point (CP) were calculated. Three parameters of SDA, namely slope of stabilogram at short-term region on log-scale SDA, slope of stabilogram at long-term region on log-scale SDA, crossover point of the short-term and long-term lines regional regression lines (*SDA*_*SSlope*, *SDA*_*LSlope*, *SDA*_*CP*) were calculated and are indicated in the Fig. 3 at the EO state.

### Analysis with sway density plot

**Fig. 4:**
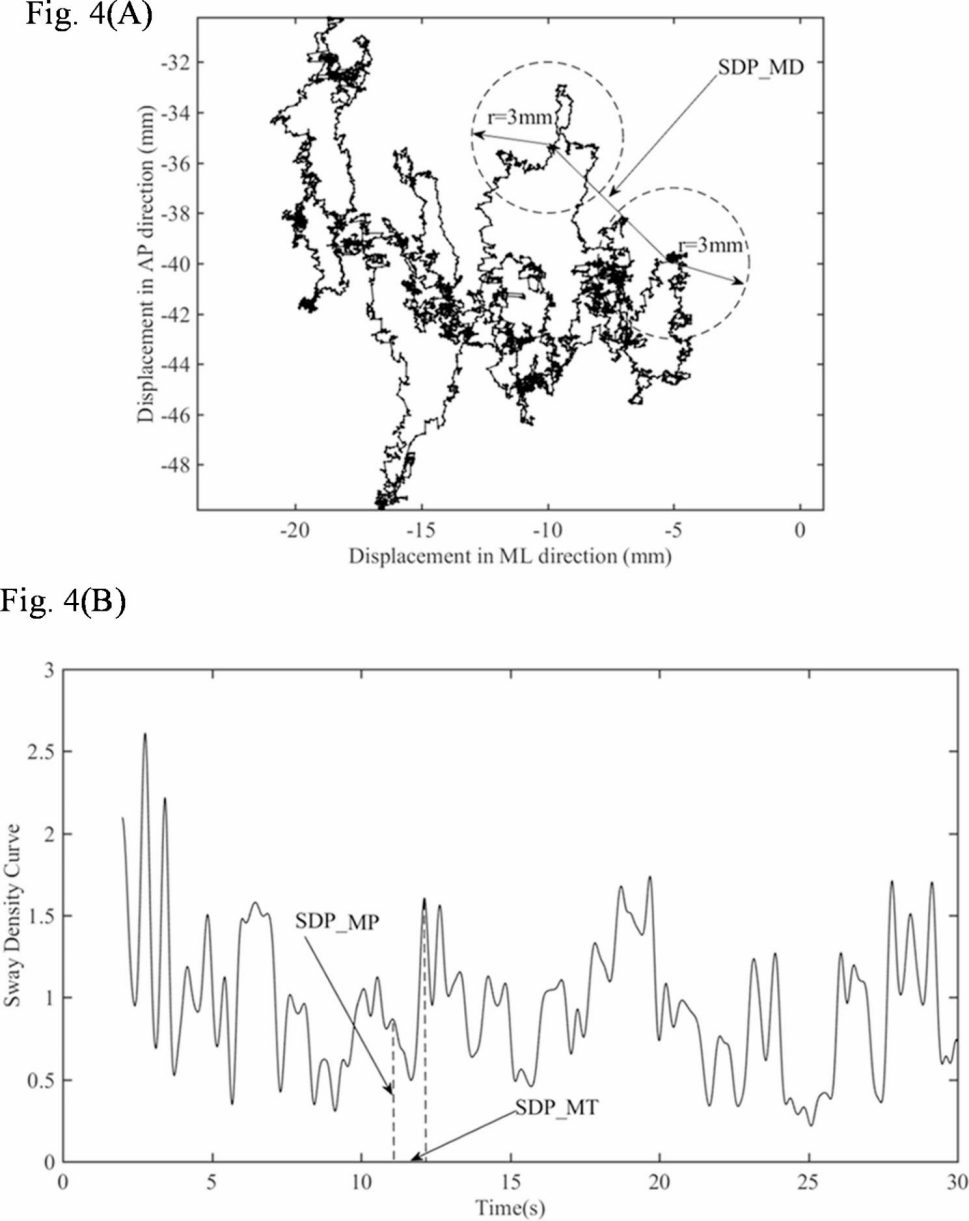
Typical SDP of a PD patient’s COP signal: (**A**) PD patient’s stabilogram; (**B**) PD patient’s sway density curve. In (**A**), R (3 mm) was the given radius within which the COP trajectory could fall instantaneously at each time interval. SDP = sway density plot, EO = Eye Open, ML = medial-lateral, AP = anterior-posterior, x = ML, y = AP, COP = center of pressure

The sway density plot (SDP) is based on a concept proposed by Baratto et al. [21]. The SDP is constructed by calculating the number of continuous samples of the COP trajectory that fell instantaneously within the given radius of the specified circle at each time interval. The given radius is 3 mm. Typical SDP shows regular peaks and troughs: the peak corresponds to the moment when the ankle torque and related motion command are relatively stable, and the valley corresponds to the moment when the ankle torque rapidly shifts from one stable value to another. Three parameters of SDP (*SDP_MP*, *SDP_MD*, *SDP_MT)* were depicted in the Fig. 4. The mean value of peaks (*SDP_MP*) was the mean value of the peaks (number of samples) in SDP. The mean value of distances (*SDP_MD*) was the mean displacement between successive peaks, and the mean value of time interval (*SDP_MT*) was the mean time interval between successive peaks.

### Statistical analysis

IBM SPSS statistics 25.0 was used for statistical analysis. Baseline characteristics and postural parameters were summarized using either means and standard deviations (SD), or frequencies and percentages as appropriate. Shapiro-Wilk test was performed to evaluate the normality of demographics and posturographic parameters in each group. The non-parametric Spearman correlation was used to calculate the correlation between each variable with BBS values. The paired sample t test or Mann-Whitney test was used to compare posturographic differences between faller with non-faller. A P value < 0.05 was considered statistically significant. To determine the sample size, a power analysis was performed based on the previously published studies [46]. A sample size of at least 45 subjects was identified to detect an effect size of 0.5 with a power of 0.8.

## Results

Correlations between posturographic parameters with BBS were listed in Table 3. Among the time domain global variables, the prediction ellipse area that encompasses 95% of COP points (*COP_PEA*) had the highest correlation with BBS at EO state (spearman’s rank correlations coefficient rho=-0.553, P value = 0.001). Among the frequency domain variables, the slope of the regression line at a high-frequency band of PSD in the medial-lateral (ML) direction (*PSD_HSlope*_*x*_) was most correlated with BBS at EO state (spearman’s rank correlations coefficient rho = 0.484, P value = 0.001). With regard to the structural variables, the highest statistically significant correlation was the crossover point of the short-term and long-term regional regression lines in AP direction (*SDA_CP*_*y*_) (rho=-0.463, P = 0.002) at EO state. The second most relevant structural parameter was the population mean value of the distance between successive peaks (SDP_MD) (rho=-0.539, P = 0.001).

**Table 3:** Spearman’s rank correlations between posturographic parameters with BBS at EO state

	(mean ± SD)	rho (P value)
*COP_v*_*x*_(m/s)	0.029 ± 0.006	-0.371 (0.011^*^)
*COP_v*_*y*_(m/s)	0.044 ± 0.02	-0.24(0.106)
*COP_SD*_*x*_	0.00765 ± 0.001	-0.243(0.103)
*COP_SD*_*y*_	3.26 ± 2.34	-0.268(0.072)
*COP_PEA*(cm^2^)	35.67 ± 9.86	-0.553(0.001^**^)
*COP_Rg*_*x*_(m)	0.084 ± 0.03	-0.239(0.011)
*COP_Rg*_*y*_(m)	0.111 ± 0.03	-0.137(0.364)
*COP_SP*_*x*_(cm)	159.77 ± 21.7	-0.211(0.159)
*COP_SP*_*y*_(cm)	139.11 ± 20.9	-0.203(0.177)
*PSD*_*F80*_x_(Hz)	1.14 ± 0.25	-0.444(0.001^**^)
*PSD*_*F80*_x_(Hz)	1.35 ± 0.3	-0.055(0.715)
*PSD*_*LPeak*_x_(Hz)	1.34 ± 0.19	-0.103(0.496)
*PSD*_*LPeak*_y_(Hz)	0.31 ± 0.12	-0.206(0.17)
*PSD*_*HPeak*_x_(Hz)	0.45 ± 0.23	-0.29(0.047^*^)
*PSD*_*HPeak*_*y*_(Hz)	0.453 ± 0.23	-0.294(0.046^*^)
*PSD_LSlope*_*x*_	-0.97 ± 0.15	0.111(0.462)
*PSD_LSlope*_*y*_	-0.43 ± 0.15	-0.072(0.636)
*PSD_HSlope*_*x*_	-0.058 ± 0.07	0.484(0.001**)
*PSD_HSlope*_*y*_	-0.1 ± 0.09	0.385(0.008^**^)
*PSD*_*CP*_x_(Hz)	0.5 ± 0.01	0.11(0.466)
*PSD*_*CP*_*y*_(Hz)	0.48 ± 0.03	0.358(0.015^*^)
*SDA_SSlope*_*x*_	1.73 ± 0.27	0.309(0.044*)
*SDA_SSlope*_*y*_	-0.43 ± 0.15	-0.072(0.636)
*SDA_LSlope*_*x*_	1.41 ± 0.06	0.309(0.04^*^)
*SDA_LSlope*_*y*_	-0.43 ± 0.15	-0.072(0.63)
*SDA* _ *CP*_*x*_	-0.97 ± 0.38	-0.073(0.63)
*SDA* _ *CP*_*y*_	-1.16 ± 0.254	-0.463(0.001**)
*SDP*_*MT*(s)	0.43 ± 0.03	-0.133(0.37)
*SDP*_*MP*(Hz)	58.2 ± 0.63	0.037(0.81)
*SDP*_*MD*(m)	0.0055 ± 0.001	-0.539(0.001^**^)

Note: ** significant at P = 0.01; * significant at P = 0.05; BBS: Berg Balance Scale; PD = Parkinson’s disease; EO: eye open; SD: Standard Deviation; rho = spearman’s rank correlation

As mentioned above, *COP*_*PEA*, *PSD*_*HSlope*_x_, *SDA*_*CP*_y_ and *SDP*_*MD* were the most relevant to BBS posturographic variables. The correlations of these four parameters with BBS under both EO and EC states were shown in Table 4 for comparison. Under the EC condition, three variables also showed significant correlations with BBS, namely, variable *COP*_*PEA* (rho=-0.349, P value = 0.019), *PSD*_*HSlope*_x_ (rho = 0.491, P value = 0.001), *SDP*_*MD* (rho=-0.385, P value = 0.007). However, the variable *SDA*_*CP*_y_ did not show correlation with BBS at EC (rho=-0.196, P value = 0.226).

**Table 4:** Spearman’s rank correlations between posturography parameters with BBS at EO and EC states

	EO		EC	
	(mean ± SD)	rho (P value)	(mean ± SD)	rho (P value)
*COP_PEA*(cm^2^)	35.67 ± 9.86	-0.55(0.001^**^)	53.94 ± 26.9	-0.349(0.019^*^)
*PSD_HSlope*_*x*_	-0.058 ± 0.07	0.484(0.001^**^)	-0.162 ± 0.048	0.491(0.001^**^)
*SDA* _ *CP*_*y*_	-1.16 ± 0.254	-0.463(0.002^**^)	-1 ± 0.73	-0.196(0.226)
*SDP*_*MD*(m)	0.0055 ± 0.001	-0.539(0.001^**^)	0.008 ± 0.002	-0.385(0.007^**^)

Note: ** significant at P = 0.01; * significant at P = 0.05; EO = Eye Open; EC = Eye Closed; COP = center of pressure; BBS: Berg Balance Scale; PD = Parkinson’s disease; SD: Standard Deviation

**Fig. 5:**
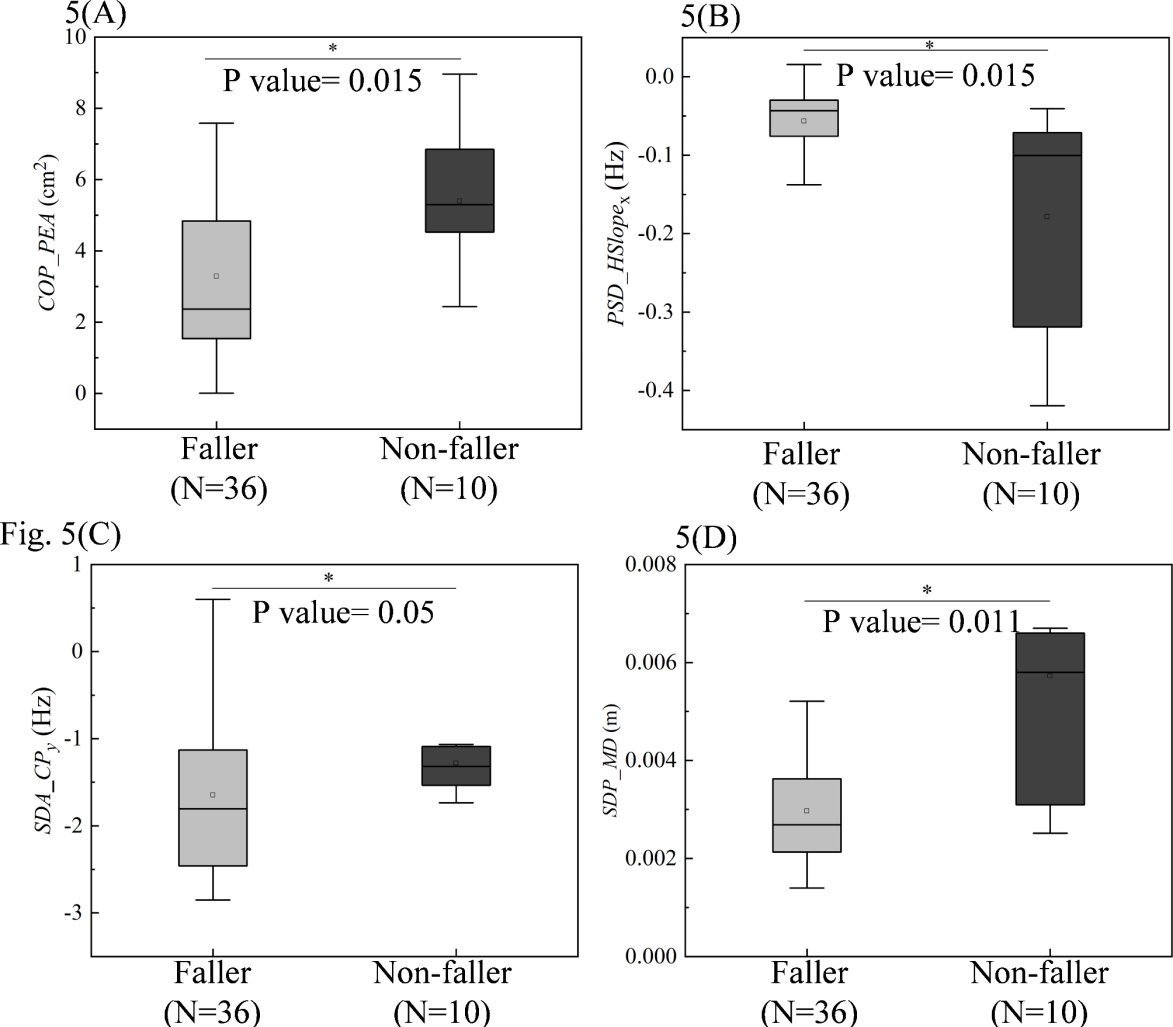
Statistic assessment of the four posturographic parameters between faller and non-faller: (**A**) *COP*_*PEA*; (**B**) *PSD*_*HSlope*_x_ ; (**C**) *SDA*_*CP*_y_ ; (**D**) *SDP*_*MD*. EO = Eye Open; COP = center of pressure, PEA = he prediction ellipse area, SDP = sway density plot, SDA = Stabilogram Diffusion Analysis, PSD = Power Spectral Density

The four correlated variables with BBS in the EC state were then applied to analyze the difference between two groups of PD patients: faller vs. non-faller (patients with/without a history of falls in the past 12 months). The four parameters were all significantly different between the faller and non-faller groups, as depicted in the Fig. 5 (*COP*_*PEA* with P value = 0.015; *PSD*_*HSlope*_x_ with P value = 0.011; *SDA*_*CP*_y_, with P value = 0.05; *SDP*_*MD* with P value = 0.015).

## Discussion

This study explored the characterization of posturographic parameters and their correlation with the functional balance ability in PD patients. Functional balance was evaluated by the clinical method of the Berg Balance Scale, while their postural stability was measured and analyzed with their COP data collected from a precise force platform.

Among the global and structural posturographic variables, several variables showed significant correlation with BBS in the state of EO, and *COP_PEA*, *PSD_HSlope*_*x*_, *SDA*_*CP*_*y*,_*SDP*_*MD* were the most correlated variables with BBS. In the state of EC, the variables *COP*_*PEA*, *PSD*_*HSlope*_x_, *SDP*_*MD* also showed significant correlation with BBS. However, in the EC state, there was no obvious correlation between *SDA_CP*_*y*_ and BBS. Visual input has predominantly influenced corrective responses for the control of AP sway in all bandwidths [47]. When the vision is blocked, one has to rely more on the somatosensory system for postural control [48]. Consequently, the sensitivity of *SDA_CP*_*y*_ was reduced without visual feedback, and its correlation with BBS was reduced. These four variables were compared between the faller and the non-faller groups, and all reached the extent of significant difference. The result was also consistent with the fact that BBS predicts the risk of falling [6].

Global COP parameters like *COP_PEA* obtained from posturography are used to assess human postural stability. For example, relatively larger sizes of area are considered signs of postural instability [49]. In our study, *COP_PEA* was the highest correlated variable with BBS and can be used as an effective indicator to assess the standing balance of PD patients.

Analysis with PSD transfers the time series of the COP position signal into frequency domains. Examining the power spectra of the COP time series provides more information about the structure of the COP signal. As shown in the results, the slope of the regional regression line of 0.5–1.2 Hz (*PSD*_*Hslope*_x_) was positively correlated with BBS in the ML direction. There was strong evidence that most of the COP power was exhibited between 0.01 and 1.2 Hz [50]. The frequencies below 0.5 Hz reflect an oscillation that was part of the descending drive to the motor neuron pool, whereas frequencies from 0.5 to 1.2 Hz likely reflected visual regulation of the motor output [51]. As the frequency band shifts from a lower to a higher frequency, the correlation with BBS increased. The frequency bandwidth of the COP signals can detect the presence of quick transients in the descending motor commands. According to the study by Kamieniarz et al., COP oscillations below 0.5 Hz are exacerbated in early and moderate PD relative to the healthy group in the state of EO. In contrast, only moderate PD patients exhibit greater power from 0.5 to 1 Hz [16]. Larger sway in each of these frequency bands reflects increased activity within the relevant postural subsystem, either due to pathology or due to compensatory efforts. The COP power in the high-frequency band increases as the BBS decreases. The increasing power could be attributed to impaired postural control in PD patients [52] and can be explained by an intermittent feedback control mode [53]. The crossover frequency of PSD was positively correlated with BBS, meaning its value decreased as BBS decreased. This can be interpreted as a result of the disappearance of antiphase coordination between ankle and hip joints in PD patients during quiet standing [52].

The results of SDA showed that the crossover point (CP) in the AP direction had a negative correlation with BBS under EO. In the diffusion diagram obtained by SDA, there were two straight fitting lines (Fig. 3) with slopes > 1 and < 1, separated by the crossover point. The corresponding slope values (*SDA_SSlope*, *SDA_LSlope*) can be used to represent the characteristics of the short-term persistent and long-term anti-persistent signals. The postural control system adopts an open-loop control scheme associated with persistence, while a closed-loop control scheme is associated with anti-persistent signals [30]. Recently, the theory of intermittent control has arisen as an attractive supplemental mechanism for postural control. It suggests that ballistic, preprogrammed signals provides slow-scale central executive control driving a fast-scale, continuous feedback inner control loop [54]. The COP signal is persistent on a fast scale, meaning that the COP signal either continues to decrease until movement is stopped or continues to increase until it became anti-persistent, followed by a decrease to draw back the COP from moving outside of the reference frame, and vice versa [55]. The size of CP represented the sensitivity of postural stability adjustment, that is, the threshold value for intermittent active postural regulation [56]. The smaller the CP values the more sensitive of regulation. The *SDA_CP* result indicates that PD patients with higher BBS scores would more actively adjust their postural stability. This is consistent with the results given by Perera et al. that the frequency of PD patients’ intermittent adjustment was positively correlated with balance ability [57].

The *SDP* supposedly decomposes the motion control into a series of motion commands, and its variables reflected the ability of the ankle joint to receive feedforward control commands in human body control [21]. The experimental results showed that *SDP_MD* was negatively correlated with BBS in the EO state, meaning that the spacing between *SDP* peaks increased as BBS decreased and indicating a decreasing frequency of patients receiving feed-forward signals. As previously mentioned in the intermittent control theory, preprogrammed command is very important in postural control, and a decreasing frequency of receiving feed forward indicates reduced postural control activity, which is often resulted from impairment of the Central Nervous System (CNS). Jacono et al. studied the relationship between *SDP* and the underlying postural stabilization process, and found that *SDP* is associated with the critical ankle torque component for overall stability of the human’s standing posture [22].

The strongest correlation presented was the variable *COP*_*PEA*, (r=-0.55)which was only mid-strong if only the correlation coefficient was considered. This study pursued to correlate the posturographic variables with the total BBS score as commonly applied in clinical diagnosis. The BBS has 14 items and their roles in the balance assessment are different. BBS items related to single leg standing (from 13 to 14) are the most distinctive evaluation items for assessing PD patients, whereas items related to double legs (from 1 to 12) are low distinguishable [58]. The patients’ COP data were obtained through double legs standing on the force platform. The different physical balance conditions will inherently lead to weak correlations between the measured results by two methods. The correlation analysis with BBS items was listed in the supplemental material.

The current study revealed that the global posturographic variable *COP*_*PEA* was statistically correlated with BBS in PD patients and can potentially be applied as quantitative indicators of balance ability in PD patients. In addition, frequency analysis and structural analysis by PSD, SDA, and SDP revealed that many variables obtained from the analyses were also statistically correlated with BBS. Since the frequency analysis and structural analysis reflect corresponding postural control mechanisms, the increase or decrease in these variables may imply enhancement or impairment of postural control in PD patients. Moreover, different stages of PD result in different balance ability. In the future research, the comparison of the balance ability between different stages of PD shall be studied. Although the subjective posturographic measurements cannot replace the observation scaling method in clinic applications, it still has merits as a supplemental diagnostic measure. The findings demonstrated that some parameters which have most correlated with BBS may be given particular attention during clinical practice. There exist certain limitations in the current study, for example, intra-subject variability of posturographic parameters (such as COP_PEA) was not performed, which may affect the accuracy of the statistical results. Also, the place of foot may cause variation of the standing position and affect obtained COP signal for each subject.

## Conclusions

This study confirmed that the Berg Balance Scale is associated with some posturographic parameters of PD patients during quiet standing. The findings demonstrate that some posturographic parameters could be applied as indices for the assessment of PD patients balance ability and fall risk. The moderate correlation between some of the structural variables and BBS can be explained by the postural control mechanisms involved in the structural analyses. These indices would be helpful for the clinical diagnosis of balance ability in PD patients.

### Electronic supplementary material

Below is the link to the electronic supplementary material.

Supplementary Material 1

## Data Availability

The data presented in the study are deposited in the Figshare website repository, accessible with the following link: 10.6084/m9.figshare.17708030.v6.
