# Supplementary material for: Correlation of balance posturographic parameters during quiet standing with the berg balance scale in patients with parkinson’s disease
Source: BMC Neurol. 2023 Oct 6;23:362. doi: 10.1186/s12883-023-03386-1 (PMC10557353; doi:10.1186/s12883-023-03386-1)
Supplement: Supplementary file 1 — Supplementary Material 1 [file 12883_2023_3386_MOESM1_ESM.docx]

Supplementary Materials

We analysed COP position and COP velocity data in the anterior-posterior direction (AP, noted as *y* axis), and medial-lateral direction (ML, noted as *x* axis). The velocity series were obtained by differentiating the position series.

The coordinate equation was as follows:

$x=x'-\overline{x}$ (1)

$y=y'-\overline{y}$ (2)

where *x* and *y* represent COP positions in ML direction and AP direction after the offset was removed respectively, *x*' and *y*' were the original COP displacements in ML and AP directions,$\overline{x}$and$\overline{y}$are the offsets in corresponding directions.

1. The mean velocity in each direction *COP_v_x_* or *COP_v_y_* was calculated as follows:

$COP\_v_{_{_{X}}}=\frac{1}{N\Delta t}\sum_{i=1}^{N} \sqrt{\left(x_{i+1}-x_{i}\right)^{2}}$ (3)

$COP\_v_{y}=\frac{1}{N\Delta t}\sum_{i=1}^{N} \sqrt{\left(y_{i+1}-y_{i}\right)^{2}}$ (4)

where *N* represented the total number of sampled COP data, and *i* specified the particular sampled data. The sample interval $\left(\Delta t\right)$ was set as 0.002s.

1. The standard deviations of COP position in ML and AP directions, expressed as *COP_SD_x_* and *COP_SD_y_  wer*e calculated as follows:

$COP\_SD_{x}=\sqrt{\frac{1}{N}\sum_{i=1}^{N} \left(x_{i}-\overline{x}\right)}$ (5)

$COP\_SD_{y}=\sqrt{\frac{1}{N}\sum_{i=1}^{N} \left(y_{i}-\overline{y}\right)}$ (6)

1. The prediction ellipse area (*COP_PEA*) was defined as the area of ellipse that encloses 95% the points of COP movement positions, and was calculated by the following steps:

$C=\left(\begin{matrix} \sigma_{11} & \sigma_{12} \\ \sigma_{21} & \sigma_{22} \end{matrix}\right)$ (7)

$\lambda_{1}=\frac{\sigma_{11}+\sigma_{22}+\sqrt{\left(\sigma_{11}-\sigma_{22}\right)^{2}+4\sigma_{12}^{2}}}{2}$ (8)

$\lambda_{2}=\frac{\sigma_{11}+\sigma_{22}-\sqrt{\left(\sigma_{11}-\sigma_{22}\right)^{2}+4\sigma_{12}^{2}}}{2}$ (9)

$\kappa=\sqrt{-2\ln\left(1-\alpha\right)}=\sqrt{-2\ln0.05}$ (10)

$COP\_PEA=\pi\times\kappa\sqrt{\lambda_{1}}\times\kappa\sqrt{\lambda_{2}}$ (11)

where$\sigma_{11}$and$\sigma_{22}$represented the variance of COP position in ML and AP direction respectively. $\sigma_{12}$and $\sigma_{21}$represented the covariance of COP position in ML and AP direction respectively. *C* was the eigenvalue of the covariance matrix. $\lambda_{1}$ denoted the first main component, and$\lambda_{2}$the second main component.$\kappa$was the confidence interval.$\kappa\sqrt{\lambda_{1}}$and $\kappa\sqrt{\lambda_{2}}$represented major and minor axes values of the confidence ellipse.

1. *COP_Rg* describes the maximum range of COP trajectory in ML and AP directions, respectively. They are obtained by the following formulas:

$COP\_Rg_{x}=max\left(\left|x_{i}\right|\right)$ (12)

$COP\_Rg_{y}=\max\left(\left|y_{i}\right|\right)$ (13)

1. The sway paths of the COP positions (*COP_SP*) in ML and AP direction were obtained by：

$COP\_SP_{x}=\sum_{\text{i=1}}^{N} \sqrt{\left(x_{i+1}-x_{i}\right)^{2}}$ (14)

$COP\_SP_{y}=\sum_{\text{i=1}}^{N} \sqrt{\left(y_{i+1}-y_{i}\right)^{2}}$ (15)

1. The sample entropy of COP positions, denoted as *COP_SE*, was calculated by the following formula:

$COP\_SE_{x}=-Ln\frac{A^{m_{x}}\left(r\right)}{B^{m_{x}}\left(r\right)}$ (16)

$COP\_SE_{y}=-Ln\frac{A^{m_{y}}\left(r\right)}{B^{m_{y}}\left(r\right)}$ (17)

Sample entropy was a measure of complexity that can be applied to time series data such as COP positions. In Equation (17), *N* was the total number of COP samples, *m* was number of sample sequence to be compared, and *r* was the tolerance for accepting matches. *Ln* was the negative logarithm.$A^{m}\left(r\right)$ was a probability that matches of length *m*+1, and$B^{m}\left(r\right)$was a probability that matches of length *m*.

Where, $A_{i}$ was number ,when sample sequence was m+1, distance between two vectors was less than r.Where, $B_{i}$ was number ,when sample sequence was m, distance between two vectors was less than r.

$B_{i}^{\left(\text{m}\right)}\left(r\right)=\frac{1}{N-m-1}B_{i}$ (18)

$B^{\left(\text{m}\right)}\left(r\right)=\frac{1}{N-m}\sum_{i=1}^{N-m} B_{i}^{m}\left(r\right)$ (19)

$A_{i}^{\left(\text{m}\right)}\left(r\right)=\frac{1}{N-m-1}A_{i}$ (20)

$A^{\left(\text{m}\right)}\left(r\right)=\frac{1}{N-m}\sum_{i=1}^{N-m} A_{i}^{m}\left(r\right)$ (21)

1. Different variables of power spectra density (PSD) were calculated by the following equations.

$PSD\_L=\sum_{m=i}^{j} m\Delta fG\left[m\right]$ (22)

$PSD\_H=\sum_{m=i}^{j} m\Delta fG\left[m\right]$ (23)

where power was the integrated area of PSD from particular spectra. $G\left[m\right]$was the discretized power spectrum density function ,$\Delta f$meant the frequency increment, $i$ and $j$ represented the lowest and highest discrete frequency values for integration, which were 0.01Hz and 0.5Hz for $Power\_L$, and 0.5Hz and 1.2Hz for $Power\_H$, respectively. Similarly,$COP\_FB80$ are frequent band, which was defined as:

$PSD\_FB80=\sum_{m=i}^{u} m\Delta fG\left[m\right]=0.8\left(Total\right.\left.Power\right)$ (24)

$PSD\_LPeak$denoted peak value of power spectral density at a low-frequency band (0.01-0.5Hz).$PSD\_HPeak$denoted peak value of power spectral density at a high-frequency band (0.5Hz-1.2Hz) . They can be obtained from:

$PSD\_LPeak=max\left(PSD\right),in\left[0.01\sim0.5Hz\right]$ (25)

$PSD\_HPeak=max\left(PSD\right),in\left[0.5\sim1.2Hz\right]$ (26)

$PSD\_LSlope$ and $PSD\_HSlope$ expressed the slopes of linear regression of plotted power spectral density function (PSD) at the low frequency band and the high frequency band, respectively. The value of the crossover point of two slopes was expressed by$PSD\_CP$.

8.$SDA\_SSlope$,$SDA\_LSlope$, and $SDA\_CP$represented short-term scale index, long-term scale index, and the crossover point in the method of SDA. The specific steps for calculation were as follows:

1. Calculating the cumulative deviation of time series of COP sway signal.

$k_{x}(i)=\sum_{i=1}^{n} [x(i)-\mathop{x}\limits^{\_} ]$ (27)

$k_{y}(i)=\sum_{i=1}^{n} [y(i)-\mathop{y}\limits^{\_} ]$ (28)

where $k(i)$was the sequence reconstruction which was divided into segments with an equal interval of p, and p was called as the scale index. The least square method　was adopted to carry out 2nd order polynomial fitting for each segment, denoted as $k_{p}(i)$. Calculating the fluctuation of the cumulative time series as follows, where x meant ML direction , y meant AP direction.

$F(p)=\sqrt{\frac{1}{n}\sum_{i=1}^{n} (k(i)-k_{p}(i))^{2}}$ (29)

Since *k(i)* was a time series with self-similarity characteristics, there was a power law relationship between the mean value *F(p)* of the wave function and the scale index *p*, which could be expressed as the following formula:

(30)

Where α was the scale index. $\alpha=SDA\_SSlope$, when α<1, It presented that the time series contains information related to short range.$\alpha=SDA\_LSlope$, when α>1, it presented that the time series contains information related to long range .

1. Sway Density Plot (SDP) represented changes in the time duration of how long COP trajectory stayed locally and time-continuously inside a circle with a radius of *R*, centered at a COP point at every sampling instant of time. The mean time interval between successive peaks on SDP with R=3mm, which was defined as:

$SDP\_MT=\frac{1}{k-1}\sum_{i=1}^{k-1} \left(t_{p}\left[i+1\right]-t_{p}\left[i\right]\right)$ (31)

where the instance of time for the *i*-th peak was denoted by$t_{p}\left[i\right]$, and the total number of peaks in the time-span of 60seconds was denoted by *k*. Value of the *i*-th peak of SDP by $peak\left[i\right]$,was defined as:

$SDP\_MP=\frac{1}{k}\sum_{i=1}^{k} peak\left[i\right]$ (32)

The mean distance between two COP point that correspond to the successive peaks of SDP for R=3mm.The mean distance was defined as:

$SDP\_MD=\frac{1}{k-1}\sum_{i=1}^{k-1} \sqrt{\left[x(p+1)-x(p)\right]^{2}+\left[y(p+1)-y(p)\right]^{2}}$ (33)

Supplement Table1 Correlation between the posturographic parameters and the items in the BBS one by one (at EO)

| EO | Items of Berg balance score | | | | | | | | | | | | | |
| --- | --- | --- | --- | --- | --- | --- | --- | --- | --- | --- | --- | --- | --- | --- |
| Variable | 1 | 2 | 3 | 4 | 5 | 6 | 7 | 8 | 9 | 10 | 11 | 12 | 13 | 14 |
| *COP_v_x_*(m/s) | -0.147 | / | / | -0.220 | -0.277 | -0.271 | 0.016 | -0.039 | -.308^*^ | -0.132 | -.397^**^ | -.401^**^ | -0.273 | -.324^*^ |
| *COP_v_y_*(m/s) | 0.037 | / | / | -0.058 | -0.137 | -0.064 | 0.128 | -0.053 | -0.167 | 0.025 | -0.193 | -0.262 | -0.143 | -0.241 |
| *COP_SD_x_* | -0.043 | / | / | -0.242 | -0.103 | -0.195 | 0.185 | 0.088 | -0.264 | -0.051 | -.325^*^ | -0.287 | -0.152 | -0.265 |
| *COP_SD_y_* | -0.052 | / | / | -0.048 | -0.099 | -0.038 | -0.004 | -0.107 | -0.202 | -0.127 | -0.161 | -0.254 | -0.216 | -0.268 |
| *COP_PEA*(cm^2^) | -0.242 | / | / | -0.151 | -0.278 | -0.160 | -0.040 | -0.107 | -.500^**^ | -.337^*^ | -.409^**^ | -.440^**^ | -.440^**^ | -.559^**^ |
| *COP_Rg_x_*(m) | -0.039 | / | / | -0.151 | -0.066 | -0.123 | 0.209 | 0.126 | -0.213 | 0.022 | -.325^*^ | -0.240 | -0.062 | -.302^*^ |
| *COP_Rg_y_*(m) | 0.130 | / | / | 0.095 | -0.018 | 0.131 | 0.145 | -0.180 | -0.004 | 0.022 | -0.123 | -0.086 | -0.067 | -0.126 |
| *COP_SP_x_*(cm) | -0.022 | / | / | -0.199 | -0.080 | -0.144 | 0.177 | 0.078 | -0.244 | -0.034 | -.313^*^ | -0.257 | -0.124 | -0.223 |
| *COP_SP_y_*(cm) | 0.009 | / | / | -0.091 | -0.151 | -0.099 | -0.032 | 0.078 | -0.210 | -0.006 | -0.175 | -.349^*^ | -0.120 | -0.178 |
| *PSD_FB80_x_*  (Hz) | -0.203 | / | / | -0.264 | -0.224 | -.291^*^ | -0.112 | -0.207 | -0.285 | -0.228 | -.340^*^ | -.360^*^ | -.394^**^ | -.342^*^ |
| *PSD_FB80_y_*  (Hz) | 0.024 | / | / | 0.022 | -0.080 | -0.029 | 0.169 | -0.012 | -0.007 | 0.046 | -0.122 | -0.044 | -0.015 | -0.066 |
| *PSD_LPeak_x_*  (Hz) | -0.143 | / | / | -0.199 | -0.081 | -0.207 | 0.153 | 0.151 | -.313^*^ | -0.015 | -0.177 | -0.173 | -0.083 | -0.139 |
| *PSD_LPeak_y_*  (Hz) | -0.104 | / | / | -0.264 | -0.130 | -0.205 | -0.040 | 0.063 | -.292^*^ | -0.007 | -0.179 | -.459^**^ | -0.116 | -0.174 |
| *PSD_HPeak_x_*  (Hz) | -0.190 | / | / | -0.272 | -0.203 | -.342^*^ | 0.112 | 0.053 | -.323^*^ | -0.131 | -.292^*^ | -0.291 | -0.213 | -0.272 |
| *PSD_HPeak_y_*  (Hz) | -0.009 | / | / | -0.082 | -0.100 | -0.119 | 0.257 | 0.049 | -0.118 | -0.003 | -0.174 | -0.170 | -0.046 | -0.168 |
| *PSD_LSlope_x_* | 0.147 | / | / | 0.168 | 0.099 | 0.206 | -0.145 | -0.107 | 0.261 | 0.067 | 0.266 | 0.125 | 0.126 | 0.122 |
| *PSD_LSlope_y_* | 0.065 | / | / | -0.207 | -0.245 | -0.188 | 0.128 | -0.209 | 0.053 | -0.129 | -0.210 | 0.045 | -0.048 | -0.043 |
| *PSD_HSlope_x_* | .372^*^ | / | / | 0.268 | .304^*^ | 0.269 | -0.008 | 0.024 | .451^**^ | .365^*^ | .470^**^ | .301^*^ | .451^**^ | .496^**^ |
| *PSD_HSlope_y_* | 0.246 | / | / | .354^*^ | .368^*^ | 0.286 | 0.112 | 0.136 | .424^**^ | 0.259 | .382^**^ | .389^**^ | .295^*^ | .409^**^ |
| *PSD_CP_x_*  (Hz) | 0.220 | / | / | 0.037 | -.291^*^ | 0.033 | -0.020 | -0.148 | 0.090 | 0.152 | 0.109 | -0.094 | 0.180 | 0.166 |
| *PSD_CP_y_*  (Hz) | 0.220 | / | / | .320^*^ | 0.230 | 0.258 | -0.048 | 0.248 | .436^**^ | 0.278 | .340^*^ | .307^*^ | .347^*^ | 0.235 |
| *SDA_SSlope_x_* | 0.154 | / | / | 0.250 | 0.079 | 0.207 | 0.089 | 0.065 | 0.197 | 0.089 | 0.209 | 0.295 | 0.289 | 0.283 |
| *SDA_SSlope_y_* | 0.065 | / | / | -0.207 | -0.245 | -0.188 | 0.128 | -0.209 | 0.053 | -0.129 | -0.210 | 0.045 | -0.048 | -0.043 |
| *SDA_LSlope_x_* | 0.154 | / | / | 0.250 | 0.079 | 0.207 | 0.089 | 0.065 | 0.197 | 0.089 | 0.209 | 0.295 | 0.289 | 0.283 |
| *SDA_LSlope_y_* | 0.065 | / | / | -0.207 | -0.245 | -0.188 | 0.128 | -0.209 | 0.053 | -0.129 | -0.210 | 0.045 | -0.048 | -0.043 |
| *SDA*_*CP_x_* | -0.081 | / | / | -0.237 | -0.215 | -0.184 | -0.006 | 0.141 | -0.194 | 0.012 | -.333^*^ | -.307^*^ | -0.008 | 0.030 |
| *SDA*_*CP_y_* | -0.213 | / | / | -.334^*^ | -0.237 | -0.211 | -0.223 | -0.037 | -.321^*^ | -0.248 | -.426^**^ | -.445^**^ | -.348^*^ | -.463^**^ |
| *SDP*_*MT*(s) | -0.052 | / | / | 0.113 | 0.033 | 0.125 | 0.170 | 0.079 | -0.069 | -0.141 | -0.139 | 0.018 | -0.219 | -0.094 |
| *SDP*_*MP*(Hz) | 0.125 | / | / | 0.183 | 0.080 | 0.221 | 0.071 | 0.020 | 0.135 | 0.075 | -0.027 | 0.122 | 0.078 | -0.021 |
| *SDP*_*MD*(m) | -0.242 | / | / | -.307^*^ | -0.271 | -.338^*^ | 0.096 | 0.005 | -.417^**^ | -0.256 | -.499^**^ | -.437^**^ | -.448^**^ | -.525^**^ |

EO: Eye open

Supplement Table2 Correlation between the posturographic parameters and the items in the BBS one by one (at EC)

| EC | Items of Berg balance score | | | | | | | | | | | | | |
| --- | --- | --- | --- | --- | --- | --- | --- | --- | --- | --- | --- | --- | --- | --- |
| Variable | 1 | 2 | 3 | 4 | 5 | 6 | 7 | 8 | 9 | 10 | 11 | 12 | 13 | 14 |
| *COP_v_x_*(m/s) | -0.194 | / | / | -0.160 | -0.182 | -0.125 | -0.161 | 0.053 | -.306^*^ | -0.121 | -.342^*^ | -.404^**^ | -0.241 | -.301^*^ |
| *COP_v_y_*(m/s) | -0.067 | / | / | -0.035 | -0.064 | 0.000 | -0.112 | 0.124 | -0.241 | -0.062 | -.301^*^ | -.347^*^ | -0.225 | -.316^*^ |
| *COP_SD_x_* | -0.069 | / | / | -0.186 | -0.063 | -0.127 | -0.040 | 0.219 | -.328^*^ | -0.010 | -0.275 | -.412^**^ | -0.198 | -0.282 |
| *COP_SD_y_* | -0.192 | / | / | -0.184 | -0.220 | -0.160 | -0.169 | 0.002 | -.382^**^ | -0.113 | -.312^*^ | -.490^**^ | -.319^*^ | -.336^*^ |
| *COP_PEA*(cm^2^) | -0.162 | / | / | -0.216 | -0.206 | -0.163 | -0.153 | 0.024 | -.378^**^ | -0.090 | -0.286 | -.490^**^ | -0.287 | -.360^*^ |
| *COP_Rg_x_*(m) | -0.039 | / | / | -0.151 | -0.066 | -0.123 | 0.209 | 0.126 | -0.213 | 0.022 | -.325^*^ | -0.240 | -0.062 | -.302^*^ |
| *COP_Rg_y_*(m) | 0.130 | / | / | 0.095 | -0.018 | 0.131 | 0.145 | -0.180 | -0.004 | 0.022 | -0.123 | -0.086 | -0.067 | -0.126 |
| *COP_SP_x_*(cm) | -0.022 | / | / | -0.199 | -0.080 | -0.144 | 0.177 | 0.078 | -0.244 | -0.034 | -.313^*^ | -0.257 | -0.124 | -0.223 |
| *COP_SP_y_*(cm) | 0.009 | / | / | -0.091 | -0.151 | -0.099 | -0.032 | 0.078 | -0.210 | -0.006 | -0.175 | -.349^*^ | -0.120 | -0.178 |
| *PSD_FB80_x_*  (Hz) | -0.080 | / | / | -0.067 | -0.049 | -0.041 | -0.032 | 0.005 | -0.120 | -0.053 | -0.184 | -0.188 | -0.088 | -0.160 |
| *PSD_FB80_y_*  (Hz) | 0.056 | / | / | 0.030 | 0.139 | 0.100 | 0.008 | 0.095 | 0.077 | 0.051 | 0.001 | 0.061 | 0.115 | -0.038 |
| *PSD_LPeak_x_*  (Hz) | -0.043 | / | / | -0.181 | -0.132 | -0.129 | -0.060 | 0.190 | -.360^*^ | 0.000 | -0.239 | -.382^**^ | -0.182 | -0.286 |
| *PSD_LPeak_y_*  (Hz) | -0.134 | / | / | -0.134 | -0.209 | -0.188 | -0.004 | 0.160 | -.376^*^ | -0.028 | -.332^*^ | -.466^**^ | -0.244 | -0.266 |
| *PSD_HPeak_x_*  (Hz) | -0.043 | / | / | -0.181 | -0.132 | -0.129 | -0.060 | 0.190 | -.360^*^ | 0.000 | -0.239 | -.382^**^ | -0.182 | -0.286 |
| *PSD_HPeak_y_*  (Hz) | -0.134 | / | / | -0.134 | -0.209 | -0.188 | -0.004 | 0.160 | -.376^*^ | -0.028 | -.332^*^ | -.466^**^ | -0.244 | -0.266 |
| *PSD_LSlope_x_* | -0.009 | / | / | 0.149 | 0.288 | 0.189 | -0.108 | -0.017 | 0.219 | 0.007 | 0.201 | 0.235 | 0.095 | 0.142 |
| *PSD_LSlope_y_* | 0.134 | / | / | -0.035 | 0.106 | 0.097 | -0.020 | 0.129 | 0.230 | 0.025 | 0.023 | 0.025 | 0.185 | 0.122 |
| *PSD_HSlope_x_* | .369^*^ | / | / | .469^**^ | 0.266 | .330^*^ | 0.213 | -0.019 | .419^**^ | .322^*^ | .524^**^ | .437^**^ | .415^**^ | .498^**^ |
| *PSD_HSlope_y_* | .303^*^ | / | / | .376^*^ | .329^*^ | .360^*^ | 0.237 | -0.019 | .348^*^ | 0.251 | .462^**^ | .482^**^ | 0.253 | .344^*^ |
| *PSD_CP_x_*  (Hz) | 0.104 | / | / | 0.173 | -0.066 | -0.009 | 0.213 | 0.097 | 0.031 | 0.081 | 0.182 | 0.160 | 0.121 | 0.095 |
| *PSD_CP_y_*  (Hz) | 0.009 | / | / | 0.134 | -0.024 | -0.049 | 0.092 | -0.083 | 0.041 | 0.034 | 0.245 | 0.203 | 0.100 | 0.165 |
| *SDA_SSlope_x_* | 0.160 | / | / | 0.006 | 0.008 | 0.028 | 0.039 | -0.010 | 0.159 | 0.152 | 0.196 | 0.149 | 0.157 | 0.145 |
| *SDA_SSlope_y_* | 0.077 | / | / | -0.018 | -0.057 | -0.047 | 0.007 | 0.170 | 0.048 | 0.098 | 0.081 | 0.035 | 0.074 | 0.237 |
| *SDA_LSlope_x_* | 0.250 | / | / | 0.258 | 0.138 | 0.227 | 0.072 | 0.149 | 0.185 | 0.281 | 0.132 | 0.207 | 0.222 | 0.193 |
| *SDA_LSlope_y_* | -0.034 | / | / | -0.067 | -0.227 | -0.033 | -0.222 | -0.206 | -0.135 | -0.015 | -.316^*^ | -0.223 | -0.060 | -0.264 |
| *SDA*_*CP_x_* | -0.109 | / | / | -0.175 | -.370^*^ | -0.202 | -0.234 | -0.216 | -0.138 | -0.303 | -0.267 | -0.166 | -0.211 | -0.194 |
| *SDA*_*CP_y_* | -0.052 | / | / | 0.036 | -0.256 | -0.180 | 0.033 | 0.070 | -0.218 | 0.015 | -0.274 | -.414^**^ | -0.202 | -0.150 |
| *SDP*_*MT*(s) | -0.168 | / | / | -0.122 | -0.165 | -0.007 | -.304^*^ | -0.149 | -0.076 | -0.212 | -0.037 | 0.022 | -0.123 | -0.009 |
| *SDP*_*MP*(Hz) | -0.248 | / | / | -.387^**^ | -0.180 | -0.185 | -0.238 | -0.110 | -0.246 | -0.188 | -.311^*^ | -0.261 | -0.190 | -0.181 |
| *SDP*_*MD*(m) | -0.259 | / | / | -0.259 | -0.222 | -0.192 | -0.173 | 0.049 | -.411^**^ | -0.162 | -.399^**^ | -.453^**^ | -.330^*^ | -.377^**^ |

EC: Eye close.
